# Pneumococcal conjugate vaccine induced IgG and nasopharyngeal carriage of pneumococci: Hyporesponsiveness and immune correlates of protection for carriage

**DOI:** 10.1016/j.vaccine.2017.05.088

**Published:** 2017-08-16

**Authors:** John Ojal, Laura L. Hammitt, John Gaitho, J. Anthony G. Scott, David Goldblatt

**Affiliations:** a KEMRI-Wellcome Trust Research Programme, Kilifi, Kenya; b Department of International Health, Johns Hopkins Bloomberg School of Public Health, Baltimore, MD, USA; c Department of Infectious Disease Epidemiology, London School of Hygiene & Tropical Medicine, London, United Kingdom; d Great Ormond Street Institute of Child Health, University College, London, United Kingdom

**Keywords:** Pneumococcal conjugate vaccine, Nasopharyngeal carriage, Kenya, Hyporesponsiveness, Correlates of protection

## Abstract

**Background:**

Prior studies have demonstrated hyporesponsiveness to pneumococcal conjugate vaccines (PCVs) when administered in the presence of homologous carriage. This may be substantially more important in Africa where carriage prevalence is high. Deriving a correlate of protection (CoP) for carriage is important in guiding the future use of extended PCVs as population control of pneumococcal disease by vaccination is now focused principally on its indirect effect. We therefore explored the complex relationship between existing carriage and vaccine responsiveness, and between serum IgG levels and risk of acquisition.

**Methods:**

We undertook secondary analyses of data from two previously published clinical trials of the safety and immunogenicity of PCV in Kenya. We compared responses to vaccination between serotype-specific carriers and non-carriers at vaccination. We assessed the risk of carriage acquisition in relation to PCV-induced serum IgG levels using either a step- or continuous-risk function.

**Results:**

For newborns, the immune response among carriers was 51–82% lower than that among non-carriers, depending on serotype. Among toddlers, for serotypes 6B, 14 and 19F the post-vaccination response among carriers was lower by between 29 and 70%. The estimated CoP against acquisition ranged from 0.26 to 1.93 μg/mL across serotypes, however, these thresholds could not be distinguished statistically from a model with constant probability of carriage independent of assay value.

**Conclusion:**

We have confirmed hyporesponsiveness in an equatorial African setting in both infants and toddlers. Population responses to vaccination are likely to improve with time as carriage prevalence of vaccine serotypes is reduced. We have not found clear correlates of protection against carriage acquisition among toddlers in this population. Assessing the potential of new vaccines through the use of CoP against carriage is still difficult as there are no clear-cut serotype specific correlates.

## Highlights

• We have confirmed hyporesponsiveness in an equatorial African setting in both infants and toddlers.
• Responses to vaccination are likely to improve with reducing vaccine-serotypes prevalence.
• We have not found clear correlates of protection (CoP) against carriage acquisition.
• Assessing the potential of new vaccines through the use of CoP against carriage is still difficult.

## 1 Introduction

The first pneumococcal conjugate vaccine (PCV), which contained seven serotypes, reduced the incidence of pneumococcal disease and the prevalence of nasopharyngeal carriage in both vaccinated and unvaccinated children as well as adults when introduced into routine infant immunisation programme in the USA in 2000 [1]. The indirect protective effect of PCV is caused by a vaccine-induced reduction in the risk of acquiring colonisation by vaccine serotypes (VTs), which leads to a reduction in onward transmission from young children.

Recently, data have emerged that highlight the complexity of interactions between pneumococci, the human immune system and the nasopharynx. Infants carrying serotypes 6B, 19F or 23F at the time of PCV immunisation have reduced primary IgG responses to those serotypes [2], [3] and this effect persists through to post-booster responses [4]. Rodenburg and colleagues showed that, at 24 months of age, children’s responses to PCV against these three serotypes were reduced if they had carried them at any point in the 2 years prior to vaccination [5].

PCV responses in African children are generally thought to be higher than those seen in developed country settings [6], [7], [8], [9]. For instance, the serotype-specific geometric mean fold-rise between the time of the first dose and one month after the third dose of PCV were lower in USA [7] and Finland [9] compared to South Africa [6] and The Gambia [8]. Nonetheless, in parts of Africa like The Gambia [10] and Kenya [11], [12], carriage rates from very early in life are extremely high. Given high responses to PCV it is possible that hyporesponsiveness does not occur, or is immunologically irrelevant, in equatorial Africa.

The immunological mechanism that mediates vaccine-induced protection against colonisation at the mucosal level, or against disease, is not known. While circulating IgG may have a role in preventing colonisation, as demonstrated in a mouse model in which antibody blocked colonisation through agglutination [13], local B cells producing IgG and/or IgA in the nasopharynx may also be relevant and a role for T cells has also been suggested [14], [15]. Nonetheless, to facilitate the licencing of new formulations of PCV, a single aggregate serological correlate of protection against invasive pneumococcal disease (IPD), has been derived based on circulating IgG [16], [17]. However, a recent analysis that suggested correlates of protection (CoP) for IPD vary widely by serotype [18] has questioned the biological relevance of a single aggregate CoP common to all serotypes. It is likely that, as with IPD, the CoP against carriage also vary by serotype.

Numerous assumptions were made during the development of the common serological CoP and there is equipoise in the scientific community about the relevance of the CoP to carriage and mucosal disease [19]. For some serotypes, greater concentrations of serum IgG were likely to be required to protect at mucosal surfaces (e.g. in the nasopharynx) than in blood [20]. Subsequent analysis of vaccine-induced antibody and the prevention of carriage reinforced the notion that if circulating IgG is indeed a relevant correlate for carriage, remarkably high concentrations are required to reduce carriage acquisition [21]. Deriving CoP for carriage would guide the future use of extended PCVs, as population control of pneumococcal disease by vaccination is now focused principally on its indirect effect mediated through carriage [22].

We therefore set out to explore both the relationship between existing carriage and vaccine responsiveness and between serum IgG levels and risk of acquisition by undertaking new analyses of two existing field studies of PCV in Kenya, with the following questions: (i) Does hyporesponsiveness occur in high carriage settings like Kenya? (ii) If so, can we detect this for serotypes other than the most common (e.g. 6B, 19F and 23F)? (iii) Is it possible to derive a serological correlate of protection against carriage acquisition using vaccine-induced IgG responses detected within randomized controlled trials of PCV in Kenya?

## 2 Methods

### 2.1 Data

Data from two previously published clinical trials of the safety and immunogenicity of PCV conducted in Kenya [14], [15] were further analysed in the current study. The first study (“Newborn study”) recruited 300 newborns that were randomized to receive 7-valent PCV (PCV7) in one of two vaccine schedules; at 0–10–14 weeks or at 6–10–14 weeks. The subjects received a PCV7 or 23-valent Pneumococcal Polysaccharide Vaccine (PPV23) booster dose of at 36 weeks. Serological measurements were made at 0, 6, 10, 14, 18, 36 and 37 weeks and nasopharyngeal carriage ascertained at 18 and 36 weeks. The objectives of this study were to examine the effect of a newborn vaccination schedule with PCV7 on the development of antibody and carriage prevalence. In the current analysis we used the carriage data at the time of the booster (week 36) and the serological measurements at week 36 and week 37.

The second study (“Toddler study”) recruited 600 children aged 1–4 years to examine the effect of 0, 1 or 2 doses of a 10-valent PCV (PCV10), on capsular antibody concentrations and nasopharyngeal carriage. Children were given PCV10 in three different schedules: Group A received PCV10 at day 0 and day 60; Group B received PCV10 at day 0 and day 180. Diphtheria-tetanus-pertussis (DTaP) was given as a control vaccine to group A at day 180 and to Group B at day 60. A third group, which is not considered in this analysis, received Hepatitis A virus (HAV) at day 0 and day 180 and DTaP at day 60. Antibody measurements were made at days 0, 30, 90 and 210 and nasopharyngeal carriage assessed at days 0, 30, 60, 90 and 180. Details of the study have been published elsewhere [23]. In the current analysis we used carriage data from vaccinees in Groups A and B at day 0, 60 and 180 (vaccination time points), and serological measurements 30 days post vaccination i.e. at 30, 90 and 210 days, respectively.

### 2.2 Analysis

For the newborn study, we calculated the fold-rise in serotype-specific geometric mean concentrations (GMC) between weeks 36 and 37, separately, for carriers and non-carriers for each of the seven serotypes in PCV7. The differences between the two groups (homologous carriers vs. non-carriers) were quantified as ratios of the GMC fold-rises. These ratios were derived from log-linear regression models of the booster response taking account the vaccine schedule group (6–10–14 vs 0–10–14), type of booster given (PCV7 vs PPV23) and the baseline log-concentration of IgG, at 36 weeks. Baseline IgG concentrations is adjusted for since individuals with lower concentrations have more room for greater fold-rise than individuals who already have high concentration at baseline.

For the toddler study, we pooled paired carriage data and 30-day serological responses for each of the time points of PCV10 vaccination (0, 60 and 180 days). We calculated serotype-specific fold-rises in IgG concentration 30 days later (at 30, 90 and 210 days). There were no blood samples at time 60 and 180 by design therefore we used the IgG at time 30 to adjust for responses to vaccines given at 60 and 180 days. We would expect antibody concentrations to decay from day 30 to day 60 (and from day 30 to day 180) at the same rate for subjects in both Group A and Group B; therefore, the ranks in IgG baseline between time 30 days and the time of vaccination are likely to be highly correlated, provided that natural boosting is also distributed equally in both groups. To assess the impact of carriage at the time of vaccination, GMC fold-rise ratios between homologous carriers vs. non-carriers were estimated from log-linear serotype-specific regression models of the individual level fold-rise on the carriage status, taking account of the vaccine group (Group A and B), age group (12–23, 24–35, 36–47 and 48–59 months), season (month of sample collection) and pre-vaccine (day 0 or 30) log IgG. We used Generalized Estimating Equations (GEE) to account for the correlations between the repeated measures within an individual. Data for serotypes 6B, 9V, 14, 19F and 23F were selected for the analysis since they were the most frequently carried of the 10 vaccine-type serotypes. As a supplementary analysis, we also calculated the post-vaccination GMC by pre-vaccination carriage status for both the newborn and toddler studies.

In order to derive the serotype-specific antibody threshold for vaccine efficacy against acquisition, we restricted our analysis to data from the toddler study and, in particular, to toddlers who were non-carriers at day zero. We compared carriage status at day-30 against vaccine-induced IgG concentration measured at day 30. We fitted to these two variables a model that incorporates a threshold parameter that is estimated through a profile likelihood [24], the a:b model. The model is a step-shaped function where the step corresponds to the antibody threshold. Thus, in addition to the threshold parameter, the model also contains two parameters for constant but different acquisition probabilities below and above the threshold. A test for the presence of a threshold was achieved by comparing the a:b model to a model with constant probability of acquisition independent of assay value, using a likelihood ratio test. Confidence intervals around the threshold estimates were constructed through bootstrapping.

The a:b model does not allow for adjustment of covariates, therefore, we also modelled the risk of serotype-specific acquisition as a continuous function of log-IgG concentration in a Cox proportional hazards model that accounted for age group, carriage of a heterologous serotype at the point of vaccination, log IgG on the day of vaccination and season. Non-linear relationship between the acquisition incidence and log-IgG concentration was allowed through restricted cubic splines. Having no colonisation by any serotype at day 0 predisposes one to considerably higher risk of colonisation by an index serotype relative to someone colonised by a different serotype to the index at day 0, due to serotype competition [25], [26]. This was the rationale for including carriage of a heterologous serotype at the point of vaccination in the model.

## 3 Results

In the newborn study, 235 pairs of 36- and 37-week samples were analysed. In these subjects the prevalence of carriage of PCV7 serotypes at 36 weeks ranged from 0.9% for serotype 23F to 12.8% for serotype 19F. Compared to non-carriers, the GMC fold-rise between week 36 and week 37 among carriers was substantially lower by a factor of 51–82% (Table 1). The point estimates of the GMC at post-booster (37 weeks) were higher among non-carriers at the point of vaccination, except of serotype 18C (Supplementary Table S1).

**Table 1:** Newborn study. Geometric mean fold rise between 36 and 37 weeks (with 95% confidence limits) stratified by carrier status, as well as the difference in the response between carriers and non-carriers expressed as a ratio. These ratios, and associated p values were derived from log-linear regression models of the booster response taking account of the vaccine group (EPI vs newborn), the type of booster given (Pneumococcal polysaccharide vaccine vs Pneumococcal conjugate vaccine) and log IgG in week 36.

Serotype	Carriers at 36 weeks		Non-carriers at 36 weeks		Ratio (95% CIs) for carrier/non-carrier	P-value
	n	GM fold-rise 37/36 Weeks (95% CI)	n	GM fold-rise 37/36 Weeks (95% CI)		
4	0	–	235	4.92 (4.36–5.55)	–	–
6B	6	2.85 (0.69–11.68)	229	13.52 (11.52–15.88)	0.18 (0.07–0.46)	<0.001
9V	4	1.62 (0.64–4.11)	231	5.32 (4.72–6.00)	0.31 (0.14–0.69)	0.005
14	10	1.29 (0.86–1.92)	225	2.79 (2.47–3.15)	0.49 (0.30–0.80)	0.004
18C	3	1.25 (0.87–1.79)	232	7.74 (6.87–8.73)	0.15 (0.05–0.40)	<0.001
19F	30	1.91 (1.34–2.73)	204	7.19 (6.11–8.45)	0.32 (0.21–0.48)	<0.001
23F	2	3.59 (0.02–663.49)	231	10.33 (8.80–12.14)	0.25 (0.06–1.09)	0.064

n: number of individuals.

In the toddler study, between 460 and 480 samples were analysed depending on serotype. The carriage prevalence at the time of vaccination ranged from 2.1% for serotype 9V to 8.0% for serotype 19F (Table 2). For serotypes 6B, 14 and 19F the GMC fold-rise post vaccination among carriers was lower by between 29 and 70%. For serotype 9V and 23F the GMC fold-rise were 53% and 1% higher among carriers (Table 2). Except for serotype 9V the point estimates of the GMC post-vaccination were higher among non-carriers at the time of vaccination (Supplementary Table S2).

**Table 2:** Toddler study. Geometric mean fold-rise between day 0 to 30 or day 30 to 90/210 stratified by carrier status at the time of vaccination (day 0, 60 or 180), as well as the difference in the response between carriers and non-carriers expressed as a ratio. The ratios and associated p-values were derived from log-linear serotype specific regression models, using GEE, of the individual level fold-rise on the carriage status, taking account of the vaccine group (Group A and B), age group (12–23, 24–35, 36–47 and 48–59 months), season (month of swab) and pre-vaccine (day 0 or 30) log IgG.

Serotype	Carriers at point of vaccination		Non-carriers at point of vaccination		Ratio (95% CIs) for carrier/non-carrier	P-value
	n^a^	GM fold-rise (95% CI)	n^a^	GM fold-rise (95% CI)		
6B	23	1.65 (1.22–2.24)	457	2.35 (2.14–2.59)	0.70 (0.51–0.97)	0.034
9V	10	1.88 (0.98–3.61)	466	3.06 (2.65–3.53)	1.53^b^ (0.89–2.65)	0.119
14	15	3.02 (1.99–4.58)	445	5.32 (4.65–6.10)	0.71 (0.50–1.02)	0.067
19F	38	2.12 (1.57–2.87)	439	7.61 (6.50–8.90)	0.30 (0.19–0.46)	<0.001
23F	22	3.39 (1.35–8.47)	455	4.28 (3.66–5.00)	1.01 (0.63–1.63)	0.955

a There are two repeated measures for almost all participants. These numbers reflect the number of samples rather than individuals.

b The reason why the adjusted ratio is above 1 (instead of approx. 1.88/3.06 = 0.61, which is the unadjusted ratio) is because one of the factors adjusted for (pre-vaccine log IgG) was unevenly distributed among carriers vs. non-carriers; the GMC of pre-vaccine log IgG among carriers was significantly higher at 1.61 compared to 0.49 in non-carriers. Similar case for 23F.

We computed the serological threshold for vaccine efficacy against acquisition among serotype-specific non-carriers at the first vaccination time-point (day 0) by using their titers and carriage status 30 days later in the toddler study. The estimated thresholds ranged from 0.26 to 1.93 μg/mL across serotypes, however, a test for the presence of a threshold at these points suggested no significant difference from a model with constant probability of acquisition independent of assay value (Table 3).

**Table 3:** Toddler study. The serotype-specific serological thresholds for vaccine efficacy against acquisition for five most commonly carried serotypes at day 0. The thresholds are computed using a step-shaped function where the step corresponds to the threshold with different infection probabilities below and above the threshold. The threshold with the highest profile likelihood is chosen as the parameters estimate. Confidence intervals are constructed by bootstrapping.

Serotype	Threshold (95% CI)	Carriage prevalence Ratio^a^ (95% CI)	Test for presence of a threshold^b^	Goodness of fit p-value^c^
6B	0.48 (0.07–2.68)	0.21 (0.04–0.72)	0.079	0.048
9V^d^	1.86 (1.86–22.67)	–	>0.999	0.219
14	0.26 (0.16–14.34)	0.26 (0.04–0.87)	0.542	0.851
19F	1.66 (0.85–6.60)	0.10 (0.00–0.60)	0.171	0.314
23F^e^	1.93 (0.09–1.94)	0.00 (0.00–0.00)	0.430	0.625

a Carriage prevalence ratio is the carriage risk above the threshold divided by carriage risk below threshold, the confidence interval is obtained by bootstrapping

b A likelihood ratio test for the presence of a threshold. Achieved by comparing the a:b model to a model with constant probability of infection independent of assay value. Values above 0.05 indicate no sufficient evidence of a difference in the two models at % level of significance.

c This is the Hosmer and Lemeshow goodness of fit p-value testing the null hypothesis that there is no difference between observed and model predicted values. The test assesses whether the step function represented by the a:b model is an appropriate representation of infection or whether another relationship such as a gradual one between titer and infection might be more likely than a stepped relationship. All the p-values, except that for serotype 6B, which is borderline, are above 0.05 indicating insufficient evidence against the null hypothesis at the 5% level of significance.

d There were no carriers of serotype 9V below the threshold of 1.86 mcg/ml hence the risk ratio was undefined.

e There were no carriers of serotype 23F above the threshold of 1.93 mcg/ml hence the risk ratio was zero.

**Fig. 1:**
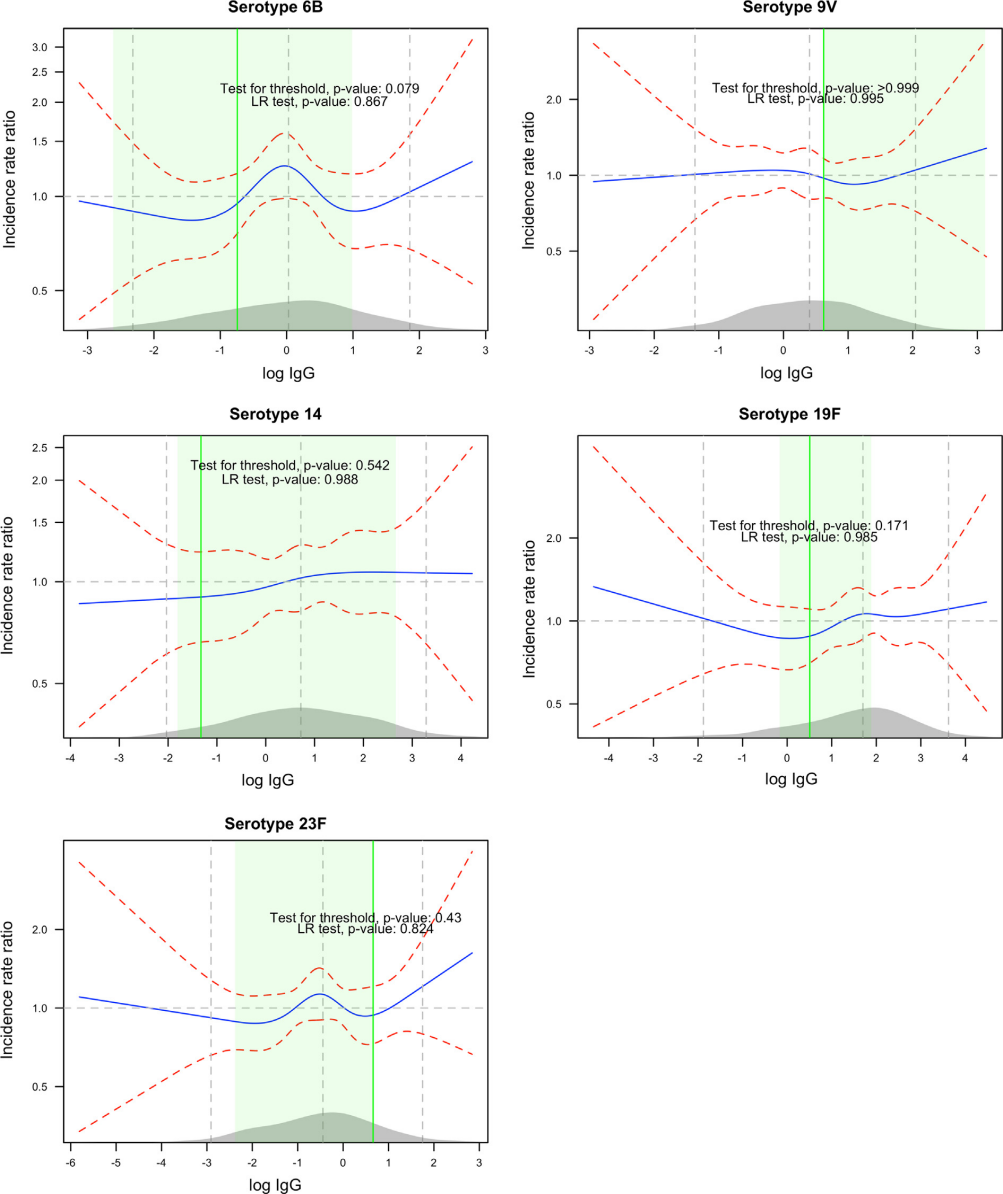
The incidence rate ratio (blue solid line) as a function of log IgG titre (x-axis) for each serotype labeled above the graph. The ratios are between the values of log IgG on the x-axis relative to someone with the average log IgG. For instance, for serotype 6B, the rate ratio between individuals with log IgG of −3 relative to individuals with the mean log IgG is slightly below 1 (95% CI: ∼0.5 to 2). The red dashed lines are the 95%CI bounds of the rate ratio. The three vertical (grey) lines mark the 2.5th, 50th and 97.5th percentiles of the distribution of log IgG whose density is shown in grey on the x-axis. The green line shows the CoP obtained by the a:b model while the light green shade around it shows the region covered by the bootstrapped 95%CI of that CoP. The likelihood ratio (LR) test p-value for the significance of log IgG in predicting carriage acquisition and the test for the presence of a threshold estimated by the a:b model is indicated in the plot. (For interpretation of the references to color in this figure legend, the reader is referred to the web version of this article.)

We analysed carriage acquisition as a continuous function of log IgG. There was no convincing monotonically decreasing rate of carriage with increasing log IgG for each of the five serotypes (Fig. 1). In a situation where a higher level IgG had strong negative impact on carriage, the prevalence ratios below the average (mean/median) log IgG would be above 1 and the prevalence ratios above the average log IgG would be below 1, in the plots.

## 4 Discussion

While inferior quantitative antibody responses to the colonising serotypes have been reported amongst children vaccinated with PCV in Philipines [2], Israel [3] and South Africa [27], none have studied this phenomenon in high carriage settings such as Equatorial Africa. Using data from two clinical trials in Kenya, we have confirmed hyporesponsiveness in equatorial Africa in both infants and toddlers, and for the first time described it in serotype 14.

The reduced immune responses to PCV administered to an individual with prevailing carriage may reduce the vaccine’s efficacy. The clinical implication of this is an increased susceptibility to acquisition of homologous pneumococcal serotypes, particularly when the reduction in immune response results in lower than sufficient protection against carriage. Several strategies can be useful in high carriage settings to counter the effect of hyporesponsiveness. The use of a catch-up campaign at the time of PCV introduction can speed-up the reduction in vaccine-type carriage thus improving the immune responses in cohorts vaccinated in the subsequent period of reduced carriage. Using a booster dose in the second year of life can also be used to overcome hyporesponsiveness [3]. However, the cost-effectiveness of such strategies needs to be evaluated to provide further evidence for or against their use.

We assessed the association between IgG concentration and the incidence of carriage in two ways; using a step function, the a:b model, which explicitly models a threshold and using a model with carriage incidence as a continuous function of IgG concentration, which does not explicitly model a threshold. The second approach allowed us to study the relation while accounting for potential confounding factors. The result from each of the approaches is mutually important and complementary in interpreting results from the alternative approach.

The CoP for carriage were generally higher than the recently derived serotype-specific CoP for IPD with the exception of serotype 14 (0.26 μg/ml for carriage acquisition vs. 0.46 μg/ml for IPD) [18]. It is expected that the CoP for carriage should be substantially higher than that for IPD; therefore, the result for serotype 14 is surprising. The evidence for the CoP for carriage being lower is, however, limited given the wide 95% confidence intervals of the CoP estimate of this serotype (Table 3) and the function of IgG that does not show drastic change around the estimated CoP (Fig. 1).

For serotype 9V, all the carriers were above the estimated CoP against acquisition. This scenario reflects one of the potential problems with the a:b model, that in the estimation process the incidence below a candidate threshold is not restricted to be higher than that above it. This requirement is, however, imposed post-estimation in the test for the existence of a threshold at the estimated value [24], such that the test statistic always yields a non-significant result in such cases. Whether circulating IgG is the correct correlate of protection also needs to be considered. The exact mechanism by which pneumococci are prevented from colonising the nasopharynx is still unclear.

The licensing of future PCVs will likely take into account the potential impact on carriage [28]. Therefore, defining the CoP for carriage would provide a way of assessing the non-inferiority of new vaccines as has been the case for CoP for IPD [16], [17]. However, until a better understanding of existing CoP for IPD exists this may be complex. For example, there is limited information to sufficiently explain why IPD correlates for some serotypes are high and others low. Consequently, predicting whether the CoP for a novel serotype will be higher or lower, and by what factor, than available CoP for other serotypes is difficult. New PCVs might incorporate serotypes that are carried relatively infrequently further complicating the use of CoP for carriage. Only one previous study that was conducted in the United Kingdom has reported on PCV CoP for carriage where a clear threshold against carriage for a single serotype, serotype 14, was identified [20]. A second study in the Navajo Nation and White Mountain Apache tribal lands, in USA, did not find identifiable IgG threshold level that was associated with prevention of carriage acquisition for all the eight serotypes studied [29].

A limitation of the newborn study is that the period between booster dose and the assessment of its effect was one week. It generally takes about 4 weeks for a full immune response following vaccination. Therefore, what we show is that the impact of pre-existing carriage on immune response is notable as early as one week. It is possible that after 4 weeks the final concentrations between carriers and non-carriers are similar. If that is the case then the effect of pre-existing carriage is in delaying immune response. From the toddler study, in which there was sufficient time-lapse between vaccination and assessment of response, the final concentrations were still different between carriers and non-carriers. It is unlikely that the case is different for newborns, because the mechanism causing hypo-responsiveness should be similar between the two age groups.

In conclusion, we have confirmed hyporesponsiveness in an equatorial African setting in both infants and toddlers. Pneumococcal conjugate vaccines have been introduced in many African countries where carriage is generally high. Hyporesponsiveness might reduce the vaccine’s effectiveness in the early years of introduction when the prevalence of vaccine serotypes is still high. If so, the speed with which vaccine-type carriage prevalence is reduced will determine how fast improved responses are realised in later years after vaccine introduction, when cohorts of children with reduced vaccine-type carriage rates replace the cohorts in high prevalence period. We did not identify clear correlates of protection against carriage acquisition among toddlers in this population. Given the limited information from the few studies that have reported on correlate of protection against carriage, assessing the potential of new vaccines through the use of correlate of protection against carriage remains difficult, as there are no clear-cut serotype-specific correlates.

## Funding

The work was supported by the Wellcome Trust fellowships [092767 to JO, 098532 to JAGS].

## Conflict of interest

LLH has received institutional research grants from Pfizer and GlaxoSmithKline. The rest of the authors do not have a commercial or other association that might pose a conflict of interest.

## Ethical approval

This paper is published with the permission of the Director, Kenya Medical Research Institute. The study was approved by the Kenya Medical Research Institute National Ethical Review Committee (SSC 2273).
