# Supplementary material for: Pneumococcal conjugate vaccine induced IgG and nasopharyngeal carriage of pneumococci: Hyporesponsiveness and immune correlates of protection for carriage
Source: Vaccine. 2017 Aug 16;35(35Part B):4652–7. doi: 10.1016/j.vaccine.2017.05.088 (PMC5571437; doi:10.1016/j.vaccine.2017.05.088)
Supplement: Supplementary data 1 [file mmc1.docx]

**Appendix**

***Table S1:*** *Newborn study. Geometric mean post-vaccination (at 37 weeks) stratified by carrier status, as well as the difference in the response between carriers and non-carriers expressed as a ratio. These ratios, and associated p values were derived from log-linear regression models of the booster response taking account of the vaccine group (EPI vs newborn), the type of booster given (Pneumococcal polysaccharide vaccine vs Pneumococcal conjugate vaccine) and log IgG in week 36.*

| Serotype | Carriers at 36 weeks | |  | Non-carriers at 36 weeks | | Ratio^a^ (95% CIs)  for carrier/non-carrier | P-value |
| --- | --- | --- | --- | --- | --- | --- | --- |
|  | n | GM at 37 weeks (95% CI) |  | n | GM at 37 weeks (95% CI) |  |  |
|  |  |  |  |  |  |  |  |
| 4 | 0 | - |  | 236 | 3.69 (3.21 – 4.24) | - | - |
| 6B | 6 | 0.71 (0.06 – 8.01) |  | 230 | 10.13 (8.44 – 12.16) | 0.18 (0.07 – 0.46) | <0.001 |
| 9V | 4 | 1.28 (0.06 – 26.16) |  | 232 | 4.04 (3.48 – 4.69) | 0.31 (0.14 – 0.69) | 0.005 |
| 14 | 10 | 4.23 (1.18 – 15.17) |  | 226 | 7.61 (6.45 – 8.98) | 0.49 (0.30 – 0.80) | 0.004 |
| 18C | 3 | 14.98 (2.52 – 89.07) |  | 233 | 3.49 (2.98 – 4.09) | 0.15 (0.05 – 0.40) | <0.001 |
| 19F | 30 | 2.25 (1.47 – 3.43) |  | 206 | 6.45 (5.50 – 7.57) | 0.32 (0.21 – 0.48) | <0.001 |
| 23F | 2 | 2.58 (0.61 – 10.95) |  | 232 | 5.49 (4.61 – 6.52) | 0.25 (0.06 – 1.09) | 0.064 |

n: number of individuals

^a^ The ratio comparing carriers vs. non-carriers obtained by regressing the log-concentration post-vaccination against carriage status at the point of vaccination (adjusting for pre-vaccination log-concentration, among other variables) is similar to that obtained when the response variable is instead the log of fold-rise (post-vaccination IgG divided by pre-vaccination IgG) and adjustment is also made for pre-vaccination log-IgG (Table 1). This is because only the coefficient of the pre-vaccination log-IgG will be altered across the two models; the coefficients of the rest of the predictors remain equal across the models.

***Table S2:*** *Toddler study. Geometric mean post-vaccination (day 30, 90/210) stratified by carrier status at the time of vaccination (day 0, 60 or 180), as well as the difference in the response between carriers and non-carriers expressed as a ratio. The ratios and associated p-values were derived from log-linear serotype specific regression models, using GEE, of the individual level post-vaccination log-IgG level on the carriage status, taking account of the vaccine group (Group A and B), age group (12-23, 24-35, 36-47 and 48-59 months), season (month of swab) and pre-vaccine (day 0 or 30) log IgG.*

| Serotype | Carriers at point of vaccination | |  | Non-carriers at point of vaccination | | Ratio^b^ (95% CIs)  for carrier/non-carrier | P-value |
| --- | --- | --- | --- | --- | --- | --- | --- |
|  | n^a^ | GM post-vaccination (95% CI) |  | n^a^ | GM post-vaccination (95% CI) |  |  |
|  |  |  |  |  |  |  |  |
| 6B | 23 | 1.03 (0.66 – 1.62) |  | 457 | 1.23 (1.12 – 1.36) | 0.70 (0.51 – 0.97) | 0.034 |
| 9V | 10 | 3.04 (1.36 – 6.77) |  | 468 | 1.51 (1.40 – 1.64) | 1.53 (0.89 – 2.65) | 0.119 |
| 14 | 15 | 2.24 (1.14 – 4.42) |  | 449 | 3.18 (2.79 – 3.63) | 0.71 (0.50 – 1.02) | 0.067 |
| 19F | 39 | 0.58 (0.32 – 1.06) |  | 442 | 7.18 (6.21 – 8.30) | 0.30 (0.19 – 0.46) | <0.001 |
| 23F | 22 | 0.74 (0.38 – 1.41) |  | 457 | 0.80 (0.72 – 0.89) | 1.01 (0.63 – 1.63) | 0.955 |

^a^There are two repeated measures for almost all participants. These numbers reflect the number of samples rather than individuals.

^b^ The ratio comparing carriers vs. non-carriers obtained by regressing the log-concentration post-vaccination against carriage status at the point of vaccination (adjusting for pre-vaccination log-concentration, among other variables) is similar to that obtained when the response variable is instead the log of fold-rise (post-vaccination IgG divided by pre-vaccination IgG) (Table 2). This is because only the coefficient of the pre-vaccination log-IgG will be altered across the two models; the coefficients of the rest of the predictors remain equal across the models.
